# A cascading-response fluorescent probe for real-time pH monitoring during cysteine-depletion process in pancreatic cancer cells

**DOI:** 10.3389/fbioe.2022.1062781

**Published:** 2022-11-03

**Authors:** Xue Qin, Shuping Zhang, Xiaolu Guo, Xingyue Liu, Xing-Can Shen

**Affiliations:** State Key Laboratory for Chemistry and Molecular Engineering of Medicinal Resources, Key Laboratory for Chemistry and Molecular Engineering of Medicinal Resources (Ministry of Education of China), Collaborative Innovation Center for Guangxi Ethnic Medicine, School of Chemistry and Pharmaceutical Sciences, Guangxi Normal University, Guilin, China

**Keywords:** cysteine depletion, pH, pancreatic cancer, imaging, fluorescent probe

## Abstract

Pancreatic cancer (PC) is one of the deadliest human malignancies, and exploring the complex molecular mechanisms behind cell death will greatly promote the clinical treatment of PC. Here, we reported a cascading-response fluorescent-imaging probe, Cy-Cys-pH, for the sequential detection of cysteine (Cys) and pH in pancreatic cancer cells. In the presence of Cys, Cys-mediated cleavage of the acrylate group caused Cy-Cys-pH to be transformed into Cy-Cys-O, which induced intense fluorescence enhancement at 725 nm. Then, Cy-Cys-O was protonated to obtain Cy-Cys-OH and the fluorescence emission shifted to 682 nm, showing a ratiometric pH response. Furthermore, Cy-Cys-pH can monitor the intracellular pH during the therapeutic process with anticancer drugs and evaluated the ability of three anticancer drugs to kill Panc-1 cells, proving that associating Cys and pH is in part an effective anticancer strategy in the treatment of pancreatic cancer. Significantly, Cy-Cys-pH is able to monitor and image pH changes during Cys depletion in real-time, which further reveals the molecular mechanism of Cys-depleted pancreatic cancer cell death, providing a powerful molecular tool for the precise treatment of PC.

## 1 Introduction

Pancreatic cancer (PC) is a near-fatal disease with disappointing outcomes owing to the difficulties in terms of early diagnosis and deep therapy (Timothy et al., 2019; Zheng et al., 2020). Therefore, an improved understanding of the molecular mechanisms and signaling pathways involved in PC is crucial for the development of new imaging and therapeutic approaches (Kelly et al., 2017; Tang et al., 2021). PC cells can upregulate metabolic programs to produce cysteine-derived metabolites that detoxify ROS to compensate reactive oxygen species (ROS) produced by mutant-KRAS signaling (Tuveson et al., 2011). Cys, a significant anti-oxidant species, is essential for regulating complicated redox balances, and increasing attention has been attracted in the regulation of cellular Cys concentration to control malignant tumor development (Snyder et al., 2018; Hughes et al., 2020; Vicente et al., 2021). Current studies have reported that it significantly suppresses the growth of pancreatic tumors in mice by depleting Cys (Olive et al., 2020). In addition, pH is also a crucial physiological parameter that affects cellular behaviors as well as other biological processes such as cell proliferation, apoptosis, and metabolism (Barber et al., 2011; Feron et al., 2017), and pH_i_ ≥ 7.2 is conducive to maintaining survival and sustaining proliferation (Dedhar et al., 2021). It is evident that breaking the alkaline intracellular pH is highly favorable for cancer therapy. Hence, to further understand the occurrence and development of PC cells, it is crucial to visualize in real-time and dynamically monitor Cys and pH in living cells.

Detections with fluorescent probes have become an ideal way for real-time monitoring of molecular events in living cells and *in vivo* (Chen et al., 2017; Yin et al., 2019; Wang et al., 2021; Ji et al., 2023). Although a variety of rationally designed fluorescent probes specifically for Cys or pH have been reported in recent years (Guo et al., 2019; Ajayaghosh et al., 2020; Kikuchi et al., 2020; Wang et al., 2020; Chen et al., 2021; Yamaguchi et al., 2021; Lin et al., 2022), the separate detection of Cys and pH in the same detection system leads to differences in probe localizations, the different metabolisms of the probes, and the failure to elucidate signaling pathways (James et al., 2021). Moreover, it might be challenging to quantify a target analyte utilizing fluorescent probes with single-emission features (Chen et al., 2022; Chen et al., 2022; Shen et al., 2022). However, there is still a lack of efficient tools for simultaneously detecting Cys and pH in living cells. It would be extremely beneficial to develop a single probe for the synchronous response of both Cys and pH to precisely reveal pH changes during the Cys depletion process in PC cell for better treatment of PC.

**SCHEME 1:**
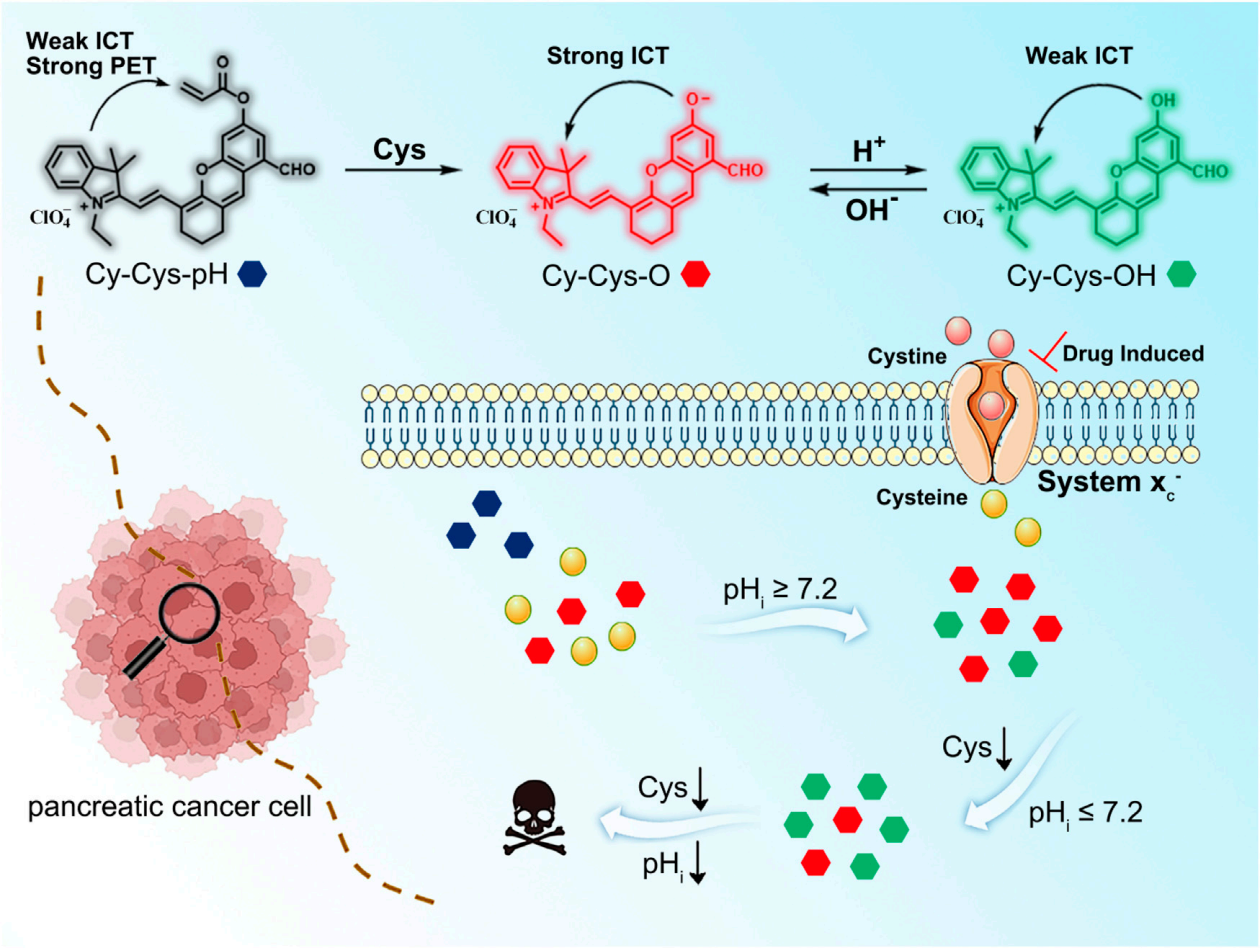
The molecular design and response mechanism of Cy-Cys-pH.

On the basis of aforementioned considerations, we have successfully designed and synthesized a cascading-response fluorescent probe, Cy-Cys-pH, with a dual-recognition site based on the combined mechanisms of photo-induced electron transfer (PET) (Supplementary Figure S1) and intramolecular charge transfer (ICT) (Supplementary Figure S2). In this probe, the hemicyanine dye was chosen as the fluorophore since it has an outstanding NIR emission characteristic and the acrylate group was chosen to be a recognition group due to its high selectivity toward Cys as well as the ability to trigger fluorescence-quenching. In the presence of Cys, Cys-mediated cleavage of the acrylate group changed Cy-Cys-pH to Cy-Cys-O, which blocked the inhibitory effect of the PET and created a strong ICT effect, exhibiting intense fluorescence at 725 nm. Then, Cy-Cys-O was protonated to obtain Cy-Cys-OH with the ICT process weakened, resulting in the fluorescence emission shifting from 725 nm to 682 nm, showing a ratio change (Scheme 1). Thus, Cy-Cys-pH made it possible for a single-molecular probe to simultaneously recognize Cys and pH as well as quantitatively monitor pH.

## 2 Materials and methods

### 2.1 Synthesis and characterization of Cy-Cys-OH

The chloro-substituted cyanine (100.0 mg, 0.16 mmol) and NaH (60% in mineral oil, 8 mg, and 0.33 mmol) were placed in a flask containing DMF (2 ml), and the mixture was stirred at room temperature under nitrogen atmosphere for 10 min. 3,5-dihydroxybenzaldehyde (43.2 mg, 0.32 mmol) in DMF (1.0 ml) was introduced to the mixture *via* a syringe, and the reaction mixture was heated at 50°C for 2 h. The solution was then removed under reduced pressure. The crude product was purified by silica-gel flash chromatography using CH_2_Cl_2_/EtOH (20: 1) as the eluent to give compound Cy-Cys-OH, a blue-green solid (35 mg, yield 41.0%).^1^H NMR (500 MHz, DMSO-*d*_6_) *δ* 10.22 (s, 1H), 8.56 (d, *J* = 15.1 Hz, 1H), 8.26 (s, 1H), 7.79 (d, *J* = 7.2 Hz, 1H), 7.72 (d, *J* = 8.0 Hz, 1H), 7.56 (t, *J* = 7.3 Hz, 1H), 7.48 (t, *J* = 7.4 Hz, 1H), 7.34 (d, *J* = 2.4 Hz, 1H), 7.17 (d, *J* = 2.2 Hz, 1H), 6.61 (d, *J* = 15.1 Hz, 1H), 4.46 (q, *J* = 7.2 Hz, 2H), 2.73 (dt, *J* = 5.8 Hz, 4H), 1.87–1.81 (m, 2H), 1.76 (s, 6H), and 1.39 (t, *J* = 7.2 Hz, 3H). ^13^C NMR (125 MHz, DMSO-*d*_6_) *δ* 193.21, 177.66, 174.83, 159.52, 154.86, 145.19, 142.76, 141.46, 132.26, 130.11, 127.74, 123.32, 119.12, 114.55, 113.66, 107.68, 105.35, 70.24, 51.02, 35.58, 31.74, 29.48, 27.67, 22.55, 20.40, 14.41, and 13.20. HRMS (ESI) calculated for C_28_H_28_NO_3_^+^ ([M]^+^): 426.2064, found: 426.2051.

### 2.2 Synthesis and characterization of Cy-Cys-pH

Compound Cy-Cys-OH (100 mg, 0.23 mmol) and Et_3_N (15 μL, 0.1 mmol) was dissolved in CH_2_Cl_2_ (10 ml), and then acryloyl chloride (41.4 mg, 0.46 mmol) was added. The reaction mixture was stirred at room temperature under nitrogen atmosphere for 3 h, and then the solution was extracted in a separatory funnel and evaporated under reduced pressure to give the crude product. The crude product was further purified by silica column chromatography with CH_2_Cl_2_/EtOH (120:1) to yield compound Cy-Cys-pH as a blue solid (65 mg, 70%). ^1^H NMR (500 MHz, DMSO-*d*_6_) *δ* 8.58 (d, *J* = 15.3 Hz, 1H), 8.21 (s, 1H), 7.86–7.78 (m, 3H), 7.72 (d, *J* = 2.3 Hz, 1H), 7.62–7.58 (m, 1H), 7.54 (t, *J* = 7.5 Hz, 1H), 6.76 (d, *J* = 15.3 Hz, 1H), 6.67–6.63 (m, 1H), 6.50 (dd, *J* = 17.3, 10.4 Hz, 2H), 6.28 (dd, *J* = 10.4, 1.0 Hz, 1H), 5.33 (t, *J* = 4.7 Hz, 1H), 4.54 (q, *J* = 7.1 Hz, 2H), 2.82–2.76 (m, 2H), 2.74–2.70 (m, 2H), 1.89–1.84 (m, 2H), 1.77 (s, 6H), and 1.67 (dd, *J* = 13.0, 6.8 Hz, 3H); ^13^C NMR (125 MHz, DMSO-*d*_6_) *δ* 192.43, 157.67, 151.76, 145.77, 143.34, 141.27, 135.24, 130.11, 127.60, 123.07, 115.25, 114.41, 107.82, 51.56, 35.59, 31.75, 29.55, 27.46, 25.58, 22.56, 14.41, and 13.48. HRMS (ESI) calculated for C_31_H_30_NO_4_^+^ ([M]^+^): 480.2169, found: 480.2161.

### 2.3 Procedure for the spectra measurement

Ultraviolet spectroscopy was carried using a Labtech UV Power PC spectrometer, and the fluorescence monitoring was done using a HITACHI F4600 fluorescence spectrophotometer. Cy-Cys-pH was dissolved in DMSO at a concentration of 1 mM as the stock solution and 30 µL of the resulting solution was transferred into a quartz fluorescence cell to obtain a final concentration of 10 µM. Then, 2 µL of a 50 mM solution of Cys was added to obtain a final Cys concentration of 0–120 μM, and the solution was mixed as the monitoring proceeded. pH titration of Cy-Cys-OH (10 µM) was performed in a DMSO/buffer solution (v/v, 3/7). The pH values were adjusted with a buffer pair. The mixture was equilibrated at room temperature for 2 min before measurement.

### 2.4 Cytotoxicity test

The *in vitro* cytotoxicity of Cy-Cys-pH was evaluated by using the standard methyl thiazolyl tetrazolium (MTT) assay in Hela and Panc-1 cells. Briefly, the cells were seeded in 96-well plates at 5,000 cells/well and cultured for 24 h. The medium was washed three times with PBS and incubated with different concentrations of Cy-Cys-pH (0, 2, 4, 6, 8, and 10 μM) for another 12 h. Then, the new medium containing 0.5 mg/ml MTT was added to each well of the 96-well assay plate and incubated for an additional 4 h. Finally, the medium washed thrice with PBS and replaced with 150 μL DMSO to dissolve the precipitates. Infinite M1000 UV-vis microplate reader (TECAN, Austria) was used to measure the absorbance at 570 nm and estimate the viability of cells.

### 2.5 Cell imaging

Panc-1 cells (1 × 10^6^) were seeded in the 35-mm glass-bottom culture dishes (Φ35 mm) and incubated for 24 h. After cells adhered to the culture dishes, the cells were washed with PBS and then incubated in culture medium supplemented with 10% fetal bovine serum; then, they were subjected to fluorescence imaging using a two-photon confocal scanning laser microscope (TCS SP8 DIVE).

## 3 Results and discussion

### 3.1 Spectral properties

**FIGURE 1:**
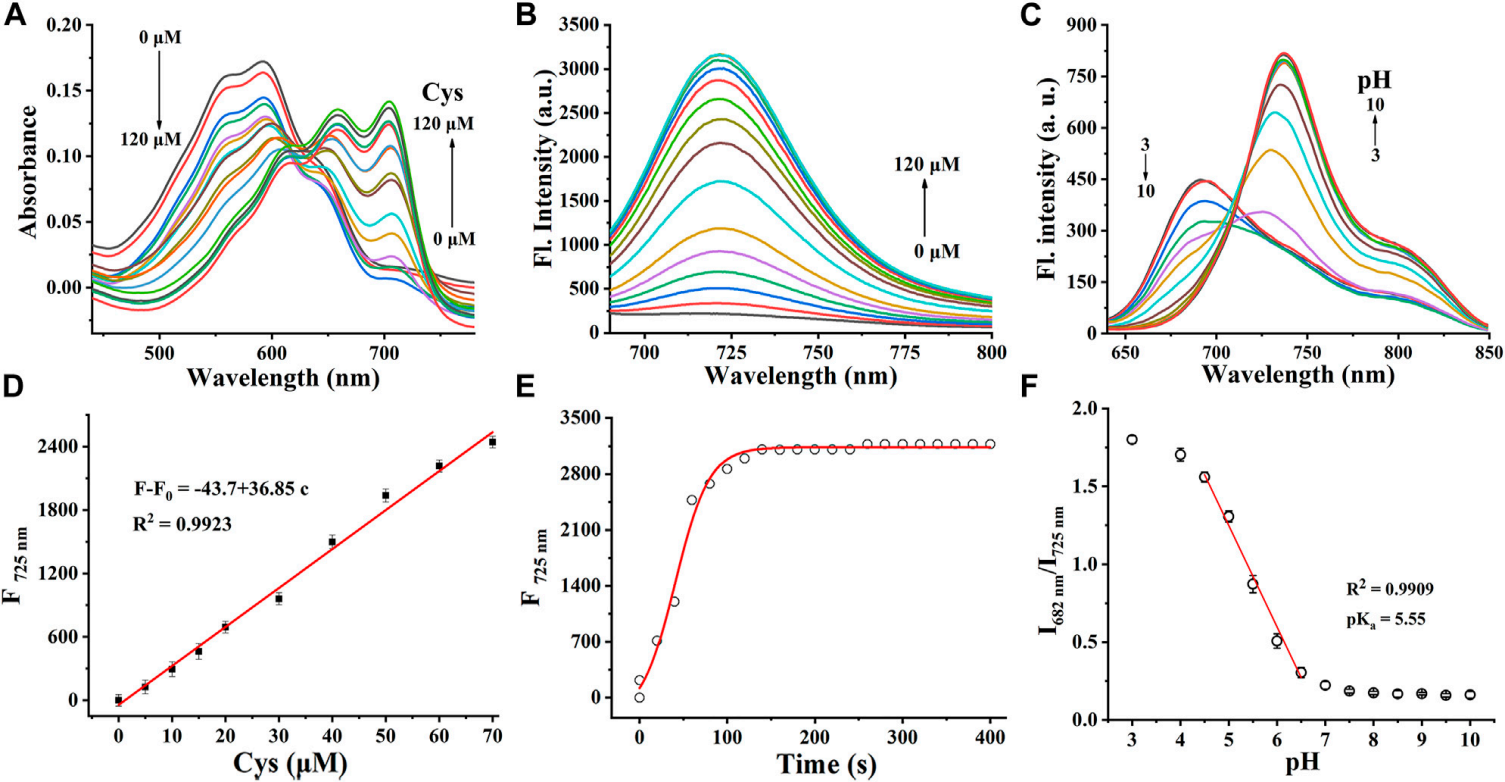
**(A)** Absorption spectra and **(B)** fluorescence responses of Cy-Cys-pH (10 μM) obtained upon addition with Cys from 0 to 120 μM. **(C)** Fluorescence spectra of Cy-Cys-OH (10 μM) in the buffer solution (pH, 3.0–10.0). **(D)** Plot of the fluorescence intensity *versus* Cys concentrations. **(E)** Time profile of Cy-Cys-pH reaction with Cys. **(F)** Plot of I_682_/I_725_ vs. pH in the range of 3.0–10.0 and the linear-fitting curve of I_682_/I_725_ vs. pH from 4.5 to 6.5. Error bar: mean ± SD, *n* = 3.

**FIGURE 2:**
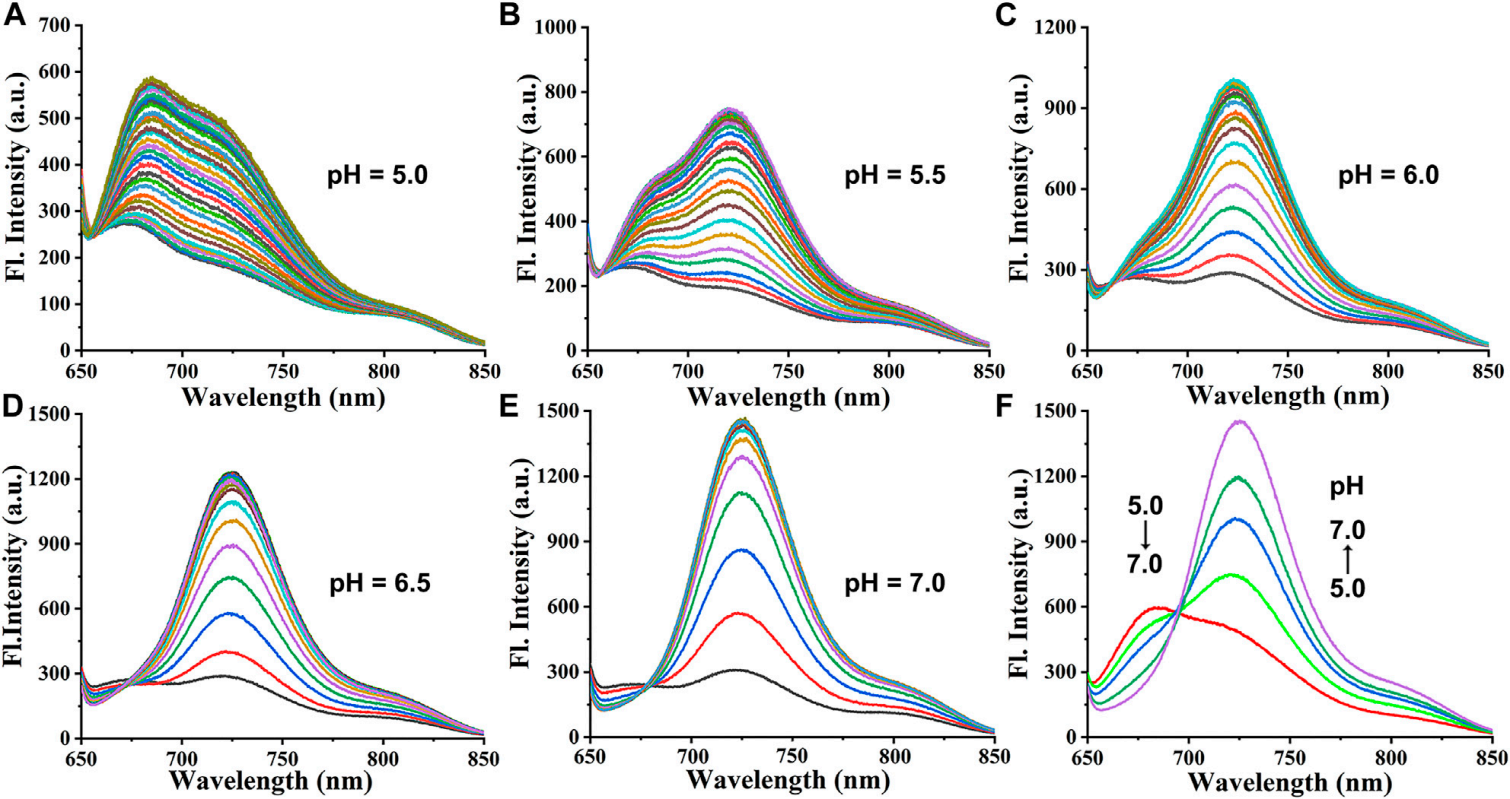
**(A–E)** Fluorescence responses of Cy-Cys-pH (10 μM) obtained upon addition with Cys (0–120 μM) in different buffer solutions (pH 5.0–7.0). **(F)** Fluorescence responses of Cy-Cys-pH (10 μM) obtained upon addition with Cys (120 μM) in different buffer solutions (pH 5.0–7.0).

Cy-Cys-OH (Shen et al., 2021) and Cy-Cys-pH were synthesized according to the synthetic route shown in Supplementary Scheme S1. Their ^1^H NMR and ^13^C NMR are exhibited in the Supplementary Figures S6–S9. First, Cy-Cys-pH was made to react with Cys to test how the absorbance spectra changed. The absorption spectra of the Cy-Cys-pH solution at various concentrations of Cys from 0 to 120 μM displayed that the absorption intensity at 592 nm steadily dropped while a new absorption peak emerged at 704 nm (Figure 1A). Meanwhile, the fluorescence intensity of Cy-Cys-pH at 725 nm gradually increased (Figure 1B), and it fit the linear relationship well between the fluorescence intensity and the Cys concentrations (Figure 1D). These findings demonstrated that at physiological Cys concentrations (30–200 µM) (Baker et al., 1990), Cy-Cys-pH was highly sensitive to Cys changes. In addition, the probe entirely interacted with Cys in 100 s and the fluorescence intensity of the mixture remained constant for at least 7 min (Figure 1E). The results proved that Cy-Cys-pH can detect Cys quickly and has good photostability. The photophysical properties of Cy-Cys-OH and Cy-Cys-pH were also examined in DMSO (Supplementary Table S1). Meanwhile, we validated the mechanism of action of Cy-Cys-pH with Cys, in which the nucleophilic addition reaction of Cys to the C=C bond of acrylate, while part of the aldehyde group, was not involved (Supplementary Figures S10, S11). Then, standard fluorescence pH titrations were performed in buffer solutions at Cy-Cys-OH concentration of 10 µM. The fluorescence emission at 682 nm decreased as the pH value increased, while it significantly increased at 725 nm, exhibiting a ratio shift (Figure 1C), and the fluorescence intensity was linear with a pH of 4.5–6.5, indicating the ability of pH-sensing activity in the tumor microenvironment pH range (Figure 1F). Furthermore, the capacity of Cy-Cys-OH to retain fluorescence intensity through the three cycles of pH 4.0 to 7.4 makes Cy-Cys-pH an excellent choice for monitoring the dynamic pH changes in the cellular system (Supplementary Table S3). When Cy-Cys-pH reacted with Cys (0–120 μM) in different buffer solutions (pH 5.0–7.0), the fluorescence intensity at 682 nm decreased and intensified at 725 nm (Figures 2A–E). This proved that the probe can respond to Cys and pH in sequence.

### 3.2 Selectivity evaluation

Before investigating the cellular experiments, we tested the sensing performance of the probe Cy-Cys-pH for Cys in the solution. The fluorescence response of the probe to various analytes, including 10 intracellular analytes (amino acids, metal ions, reactive oxygen species (ROS), and reactive nitrogen species (RNS)) is shown in Supplementary Table S4A,B. Neither fluorescence amplification nor clear interference was produced by these analytes when Cys was being detected, suggesting that Cy-Cys-pH is a reliable fluorescent probe and can work well in physiological environments.

### 3.3 Cell-toxicity experiments

The cytotoxicity of Cy-Cys-pH on Hela and Panc-1 cells was tested using the MTT assay prior to utilizing Cy-Cys-pH for imaging in cells. Even when the concentration of Cy-Cys-pH reached 10 μM, cell viability remained greater than 85%. (Supplementary Table S5). It can be inferred that Cy-Cys-pH has low cytotoxicity and is suitable for use in subsequent cell studies.

### 3.4 Cell experiments in response to endogenous/exogenous Cys

**FIGURE 3:**
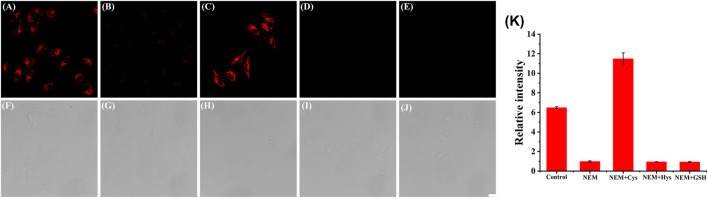
CLSM images of Panc-1 cells treated with **(A,F)** Cy-Cys-pH (5 μM), **(B,G)** NEM (10 μM), **(C,H)** Cys (100 μM) with NEM, **(D,I)** Hcy (100 μM) with NEM, and **(E,J)** GSH (100 μM) with NEM. **(K)** The corresponding histogram of the fluorescence intensities from the images in **(A–E)**. λ_ex_ = 638 nm, λ_em_ = 650–750 nm. Scale bars: 20 μm. Error bar: mean ± SD, *n* = 3.

Next, we investigated whether the probe might be used to monitor Cys in living cells. Panc-1 cells were incubated with Cy-Cys-pH at pH 7.4 in a neutral environment, resulting in considerable fluorescence, showing that Cy-Cys-pH can interact with endogenous Cys to produce fluorescence (Figure 3A). Meanwhile, there was a noticeable fluorescence quenching when the cells were treated with N-ethylmaleimide (NEM) (Figure 3B). When 100 μM Cys was added to the NEM-pretreated cells, strong fluorescence was observed once more (Figure 3C), and very little fluorescence could be induced by the Hcy and GSH treatments (Figures 3D,E). These demonstrated that Cy-Cys-pH is able to detect exogenous and endogenous Cys and is unaffected by the abundant Hcy and GSH in cancer cells.

### 3.5 Ratiometric pH fluorescence image in Panc-1 cells

**FIGURE 4:**
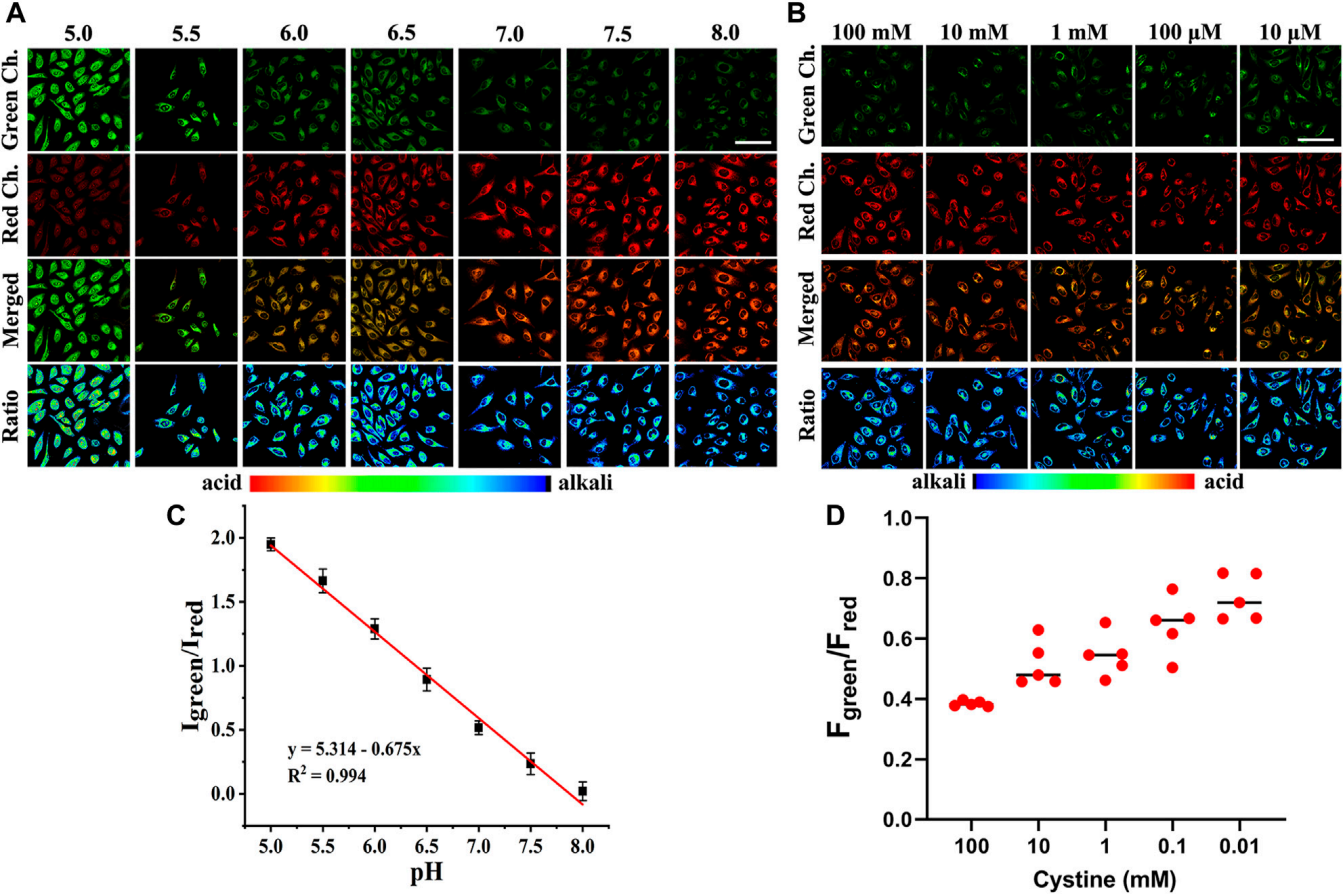
**(A)** CLSM images of Cy-Cys-OH in Panc-1 cells under different pH conditions. **(B)** CLSM images of Panc-1 cells incubated under different cystine concentrations with 5 μM Cy-Cys-pH. **(C)** The linear relationship between the ratio value I_green_/I_red_ and pH. Error bar: mean ± SD, *n* = 3. **(D)** The fluorescence intensity of green/red ratio to **(B)** under pretreatment with cystine in different concentration conditions (*n* = 5). Green Ch.: λ_ex_ = 552 nm, λ_em_ = 650–700 nm, Red Ch.: λ_ex_ = 638 nm, and λ_em_ = 700–750 nm. Scale bars: 100 μm.

Since Cy-Cys-pH first responds to Cys and then quantifies pH by the proton exchange of Cy-Cys-OH, we demonstrated the feasibility of Cy-Cys-pH in quantifying intracellular pH in living cells. Intracellular calibration experiments were conducted with Panc-1 cells in high K^+^ buffers of different pH using Cy-Cys-OH. As shown in Figure 4A, the fluorescence intensity of the red channel increased steadily, while the green channel diminished with pH from 5.0 to 8.0 following the successful establishment of a calibration curve, which demonstrated good linearity in the pH range of 5.0–8.0 (Figure 4C), indicating that the Cy-Cys-pH can effectively track intracellular pH.

### 3.6 Indicating pH changes during the depletion of Cys in Panc-1 cells

Within the cells, imported cystine *via* the cystine/glutamate anti-porter system (system X_c_^−^) is reduced to Cys in a NADPH-dependent manner to replenish intracellular Cys, and intracellular Cys decreases with reducing cystine concentrations (Mak et al., 2015). Thus, we investigated whether Cy-Cys-pH can track the dynamic pH change in cells caused by Cys depletion. As a result, we first regulated Cys by directly controlling the extracellular cystine concentration. Panc-1 cells were pre-incubated with different concentrations of cystine for 12 h and then cultured with Cy-Cys-pH for 30 min. As depicted in CLSM images (Figure 4B), when the extracellular cystine contents were reduced from 100 mM to 10 μM, the fluorescence of the green channel was relatively enhanced, while the red channel weakened, exhibiting a change in ratio. Importantly, the ratio of fluorescence intensity (F_green_/F_red_) was displayed from 0.38 to 0.73 (Figure 4D), and the intracellular pH showed a decline from 7.30 to 6.78 compared to the previously calibrated graph which clearly demonstrated that the probe was successful in tracking the dynamic pH decline along with the concentration-reduced Cys conditions.

**FIGURE 5:**
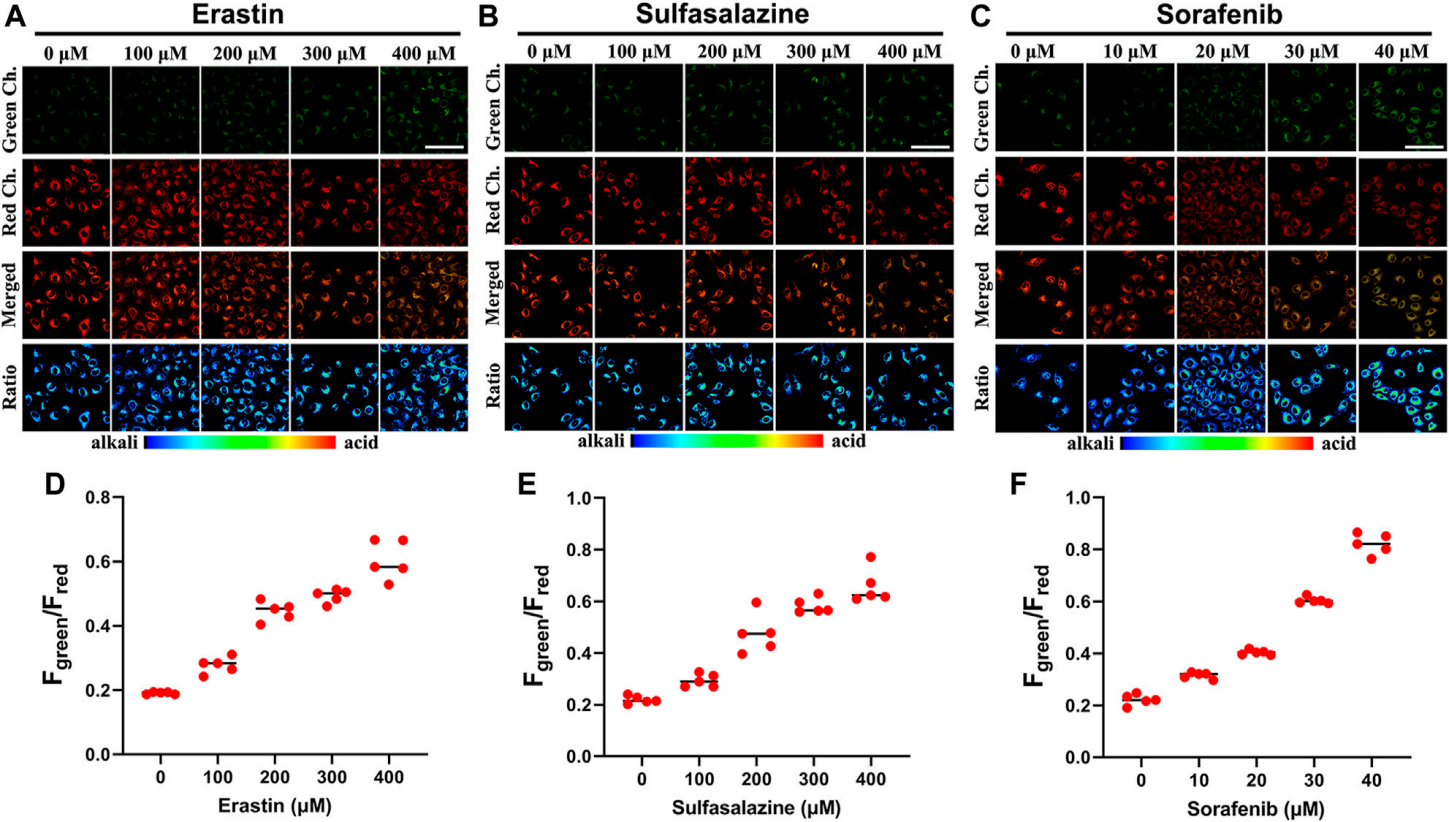
**(A–C)** CLSM images of Panc-1 cells incubated under different drug (erastin, sulfasalazine, and sorafenib) concentrations with 5 μM Cy-Cys-pH. **(D–F)** The fluorescence intensity of green/red ratio to **(A–C)** under pretreatment with drugs under different concentration conditions (*n* = 5). Green Ch.: λ_ex_ = 552 nm, λ_em_ = 650–700 nm, Red Ch.: λ_ex_ = 638 nm, and λ_em_ = 700–750 nm. Scale bars: 100 μm.

In fact, system X_c_^−^ is overexpressed in different tumor types, and the pancreatic cell line Panc-1 has high resistance to gemcitabine because it has a higher systemic X_c_^−^ expression than MIAPaCa-2 and BxPC-3, which makes it easier for cancer cells to obtain Cys through extracellular cysteine (Gout et al., 2008). In contrast, suppressing system X_c_^−^ activity is successful in preventing cystine uptake, which will result in a shortage of Cys and cause cell ferroptosis (Olive et al., 2020). Crucially, it has been found that restricting cystine uptake by inhibiting system X_c_^−^ increases the sensitivity of cancer cells to chemotherapy (Maher et al., 2013). As we all know, various anticancer drugs target cells through diverse signaling pathways, changing the pH in each condition. Simultaneously, encouraged by the aforementioned experimental findings, we explored how the intracellular pH of cells changes while being treated with various anticancer drugs. We employed three different anticancer drugs, all of which have been proven to inhibit system X_c_^−^ (erastin (Stockwell et al., 2012), sulfasalazine (Bruchovsky et al., 2001), and sorafenib (Wrosch et al., 2017)). As shown in Figures 5A,D, when Panc-1 cells were treated with erastin (0–400 μM) for 12 h and then with Cy-Cys-pH (5 μM) for 30 min, a rise in the fluorescence ratio values (F_green_/F_red_) was found and the intracellular pH decreased from 7.58 to 6.97. Similarly, the intracellular pH of Panc-1 cells was reduced from 7.54 to 6.89 after being exposed to sulfasalazine (0–400 μM) (Figures 5B,E). By comparison, cells were incubated with sorafenib at concentrations of 0–40 μM and Panc-1 cells had a decrease in the intracellular pH from 7.54 to 6.65 (Figures 5C,F), indicating that even though they are both system X_c_^−^ inhibitors, erastine and sulfasalazine are less capable of inhibiting system X_c_^−^ than sorafenib, as well as killing Panc-1 cells by depleting intracellular Cys. These results also demonstrated that Cy-Cys-pH can monitor intracellular pH during the therapeutic process with anticancer drugs. Meanwhile, the anticancer strategy by associating Cys and pH is, in part, an effective strategy as they target two features of the tumor microenvironment—oxidative stress and alkaline intracellular pH, both affecting cancer-cell progression.

## 4 Conclusion

In summary, we have put forward a cascading-response fluorescent probe, Cy-Cys-pH, with dual recognition sites. The probe can simultaneously visualize Panc-1 cells that have varied levels of Cys depletion and also monitor the corresponding intracellular pH level in real-time. The overexpressed Cys in the tumor microenvironment promoted the conversion of Cy-Cys-pH to Cy-Cys-O, resulting in a significant increase in the fluorescence emitting at 725 nm. During cell death caused by Cys depletion, the hydroxyl group in Cy-Cys-O was protonated as a result of the acidified pH, which made the emission wavelength change from 725 nm (Cy-Cys-O) to 682 nm (Cy-Cys-OH). Furthermore, Cy-Cys-pH revealed that the dynamic pH change decreased along with the concentration-reduced Cys conditions, monitored the intracellular pH during the therapeutic process with anticancer drugs, and evaluated the ability of three anticancer drugs to kill Panc-1 cells. Cy-Cys-pH can help understand the molecular mechanism of PC cell death and offers a crucial tool for the precise treatment of PC.

## Data Availability

The original contributions presented in the study are included in the article/Supplementary Material; further inquiries can be directed to the corresponding author.
