# Supplementary material for: A cascading-response fluorescent probe for real-time pH monitoring during cysteine-depletion process in pancreatic cancer cells
Source: Front Bioeng Biotechnol. 2022 Nov 3;10:1062781. doi: 10.3389/fbioe.2022.1062781 (PMC9669487; doi:10.3389/fbioe.2022.1062781)
Supplement: Supplementary file 1 [file DataSheet1.docx]

Supplementary Data

**Experimental Methods**

**Instruments and materials**

Unless otherwise stated, all reagents were purchased from commercial suppliers and used without further purification. Solvents used were purified by standard methods prior to use. Twice-distilled water was used throughout all experiments. High resolution mass spectra were acquired on an LTQ FT spectrometer. 1^H^ NMR and 13^C^ NMR spectra were recorded at ambient temperature using 500 MHz spectrometers, using TMS as an internal standard. Electronic absorption spectra were obtained on a Labtech UV Power PC spectrometer. Photoluminescent spectra were recorded at room temperature with a HITACHI F4600 fluorescence spectrophotometer with the excitation and emission slit widths at 5.0 and 5.0 nm respectively. The fluorescence imaging of cells was performed with Two-photon Confocal Scanning Laser Microscope (TCS SP8 DIVE). TLC analysis was performed on silica gel plates and column chromatography was conducted over silica gel (mesh 200-300), both of which were obtained from the Qingdao Ocean Chemicals

**Determination of the detection limit**

The detection limit (DL) was calculated based on the fluorescence titration of probe in the presence of Cys. The fluorescence intensity of probe was measured and standard deviation of the blank measurement was achieved. The detection limit was calculated by using detection limit with the following equation:

Detection limit = 3*σ*/*k*

Where *σ* is the standard deviation of the blank measurement, *k* is the slope between the fluorescence intensity versus various Cys concentrations.

**Calculation of p*K*_a_ Values**

p*K*_a_ values of the compounds were calculated by regression analysis of the fluorescence data to fit eq 1. *R* is the ratio of emission intensity at two wavelengths. *R_max_* and *R_min_* are maximum and minimum limiting values of *R*, and *c* is the slope (positive for the basic forms of the dyes and negative for the acidic forms). *I^a^/I^b^* is the ratio of the absorption intensity in acid to the absorption intensity in base at the wavelength chosen for the denominator of *R.*

$pH=pK_{a}+c\left[ log\frac{R-R_{min}}{R_{max}-R} \right]+log\frac{I^{a}}{I^{b}}$ (1)

**Theoretical computations**

To describe the ground state and singlet excited state of Cy-Cys-pH, DFT theoretical calculations were performed. All the calculations were carried out using the Gaussian 09 program package. All the geometries of Cy-Cys-pH, Cy-Cys-O and Cy-Cys-OH were optimized at B3LYP/6-31+G(d) level. The molecular orbital (MO) plots and MO energy levels were computed at the same level of theory.

**Cell culture**

Hela cells and Panc-1 cells were respective cultured in RPMI-1640 and DMEM medium supplemented with 10 % fetal bovine serum (WelGene), penicillin (100 units/mL), and streptomycin (100 μg/mL) and incubated in a humidified incubator containing 5 % CO_2_ at 37 °C.

**Synthesis and Characterization of** **Cy-Cys-OH and Cy-Cys-pH**

**Scheme S1**. Synthesis of compounds Cy-Cys-OH and Cy-Cys-pH.

**Table S1.** Photophysical Properties of Cy-Cys-OH and Cy-Cys-pH in DMSO.

| Compd. | λ_max_ (nm) | λ_em_ (nm) | Stokes shift (nm) | ε (10^4^) | Ф (%) |
| --- | --- | --- | --- | --- | --- |
| Cy-Cys-OH | 705 | 725 | 20 | 1.42 | 1.4 |
| Cy-Cys-pH | 590 | 690 | 100 | 1.72 | 7.5×${10}^{-3}$ |


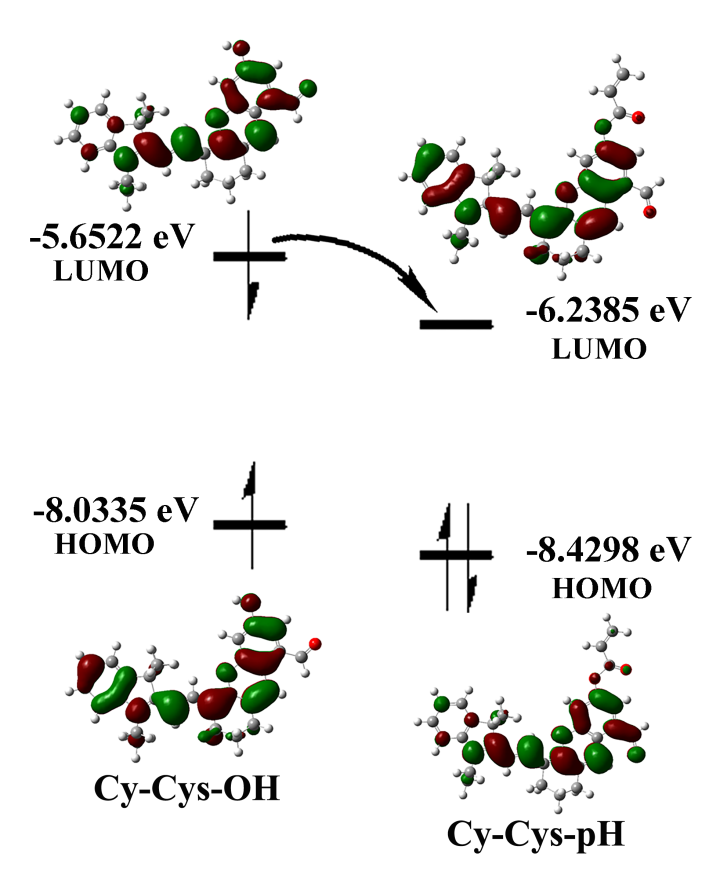


**Figure S1.** Theoretical verification of the PET mechanism for Cy-Cys-pH.


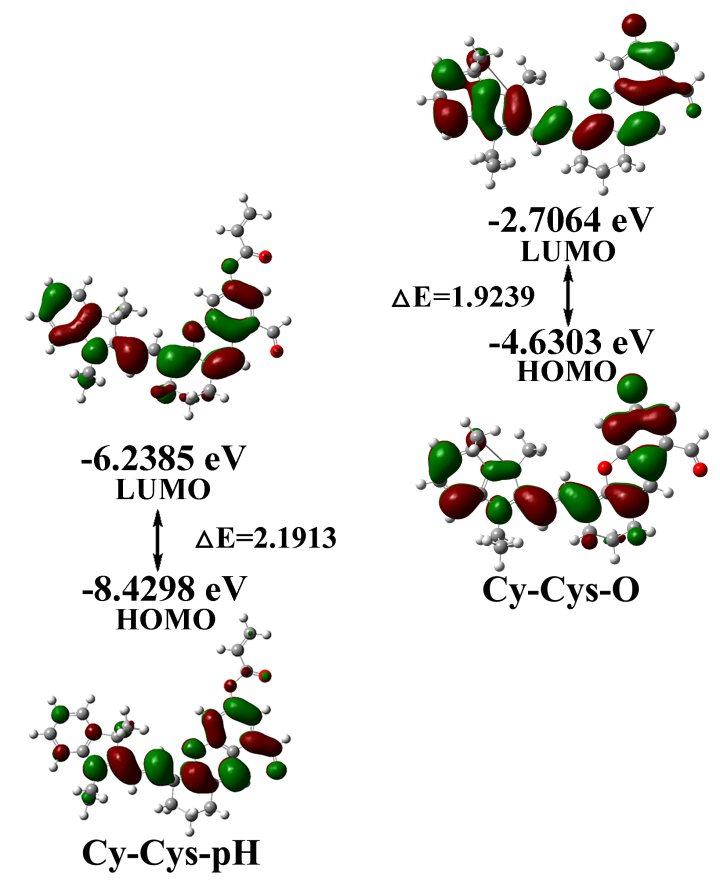


**Figure S2.** Theoretical verification of the ICT mechanism for Cy-Cys-pH.





**Figure S3.** Reversibility in the fluorescence switching of Cy-Cys-OH between pH 4.0 and 7.4.


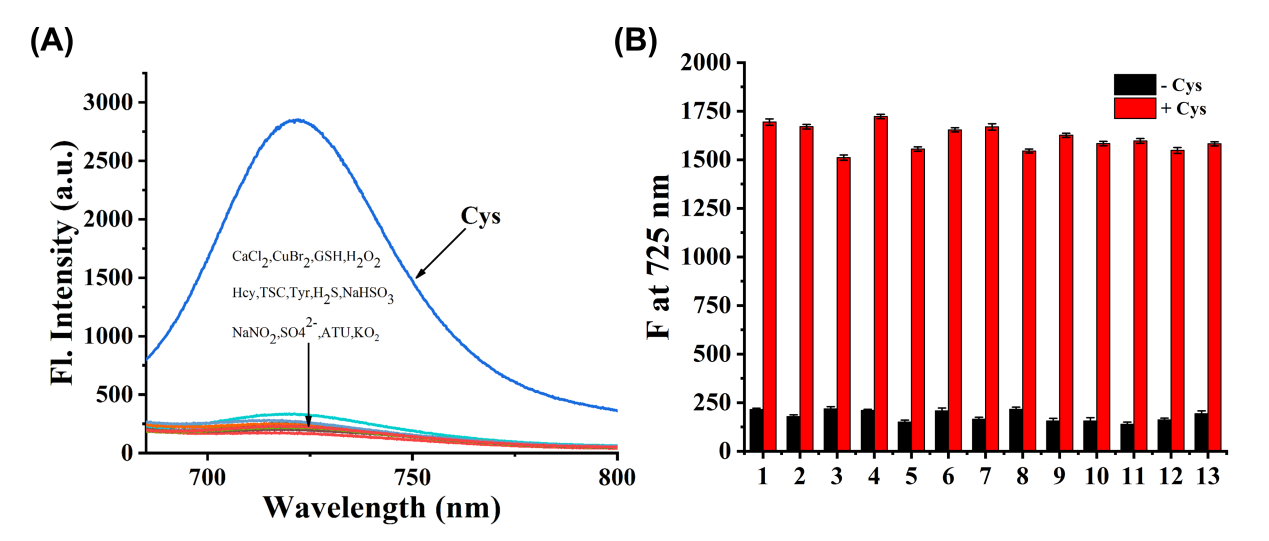


**Figure S4**. (A) Fluorescence spectra of Cy-Cys-pH (10 μM) reacted with 120 μM of various analytes. (B) Fluorescent intensity responses of Cy-Cys-pH (10 μM) to various analytes after 5 min at 725 nm. Black and red columns indicate no or extra addition of 120 μM Cys. From 1 to 13: CaCl_2_, CuBr_2_, GSH, H_2_O_2_, Hcy, TSC, Tyr, H_2_S, NaHSO_3_, NaNO_2_, SO_4_^2-^, ATU, KO_2_. Error bar: Mean ± SD, n = 3.





**Figure S5**. Cytotoxicity of the probe on Hela and Panc-1 cells determined by MTT. Error bar: Mean ± SD, n = 3.


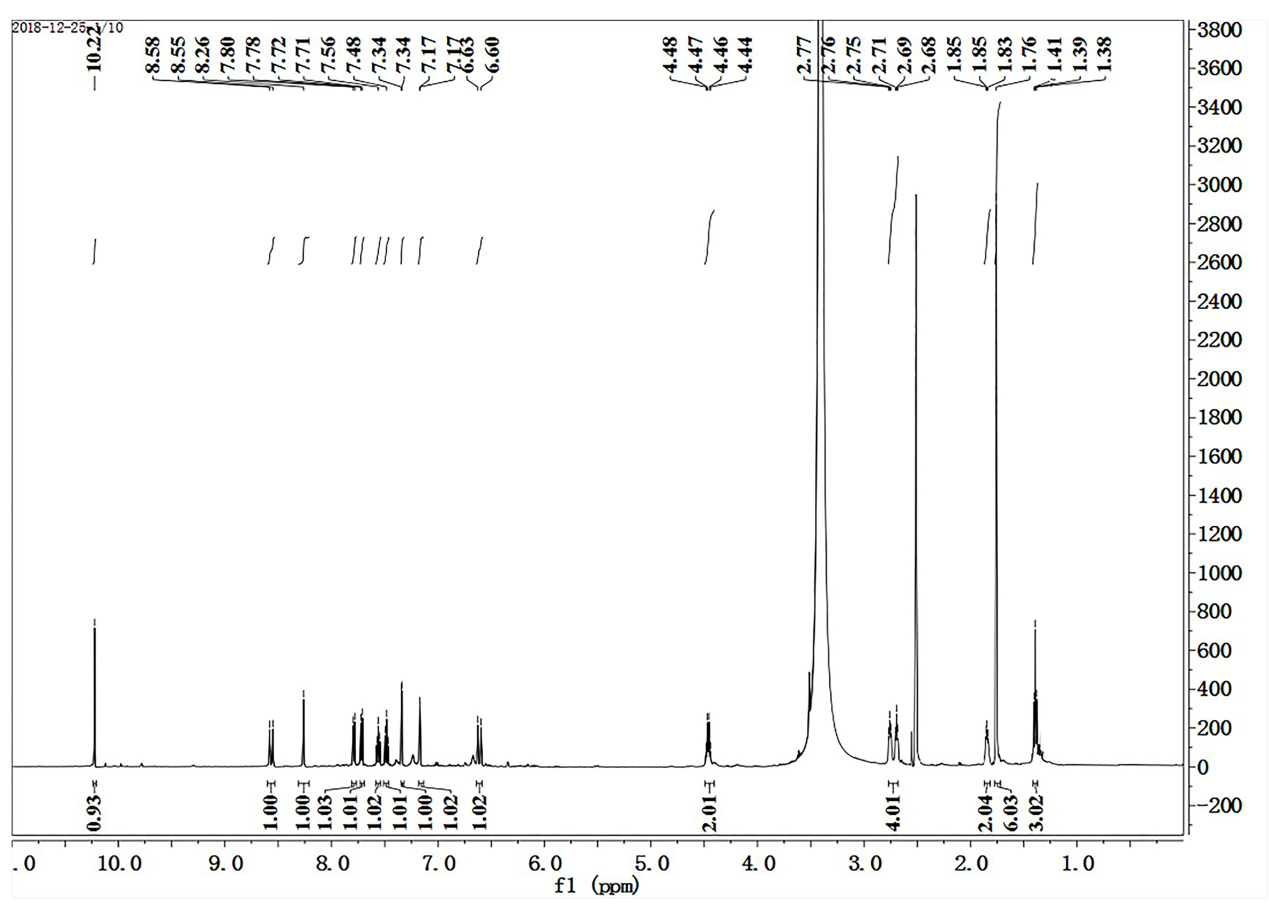


**Figure S6.** ^1^H NMR spectrum of **Cy-Cys-OH.**


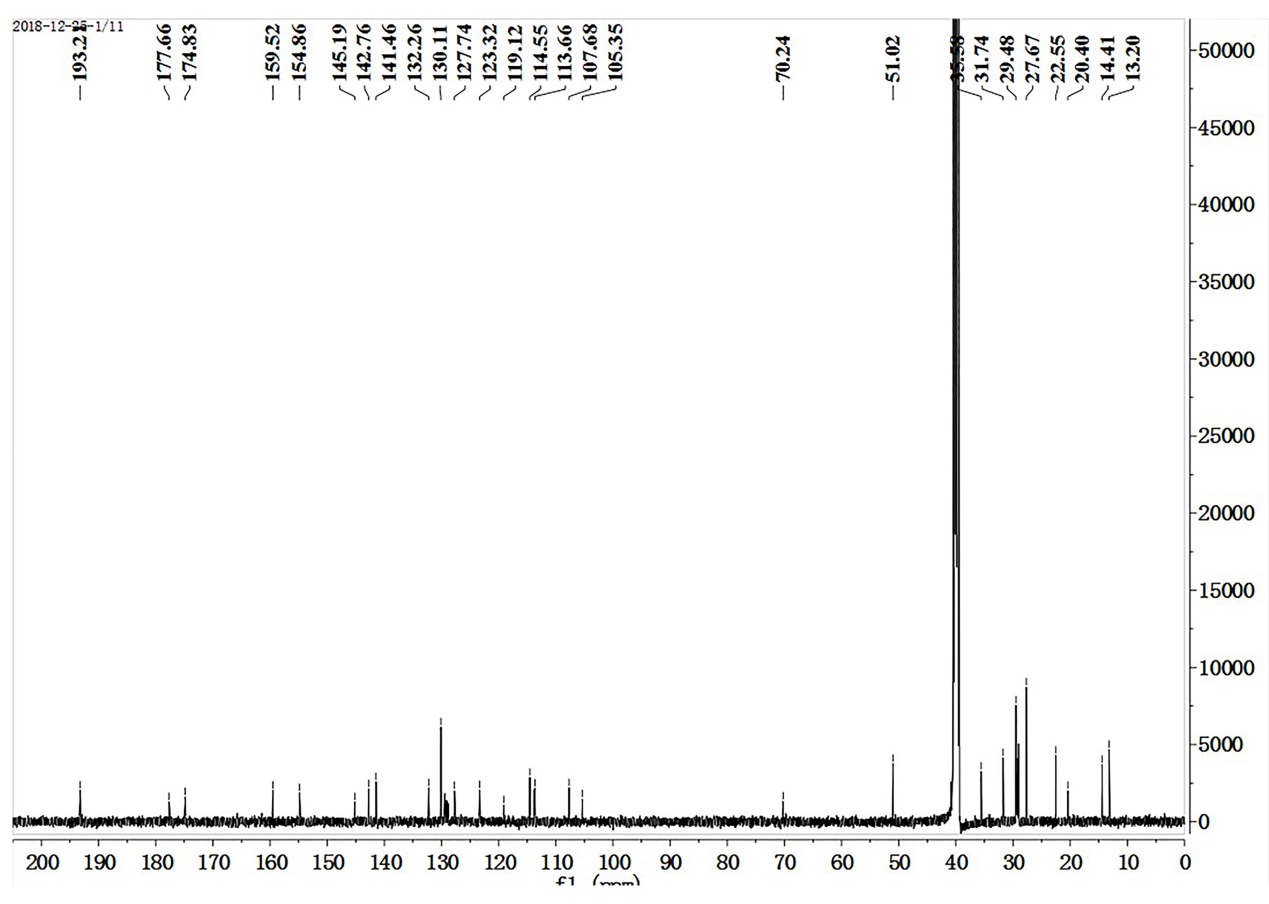


**Figure S7.** ^13^C NMR spectrum of **Cy-Cys-OH_._**


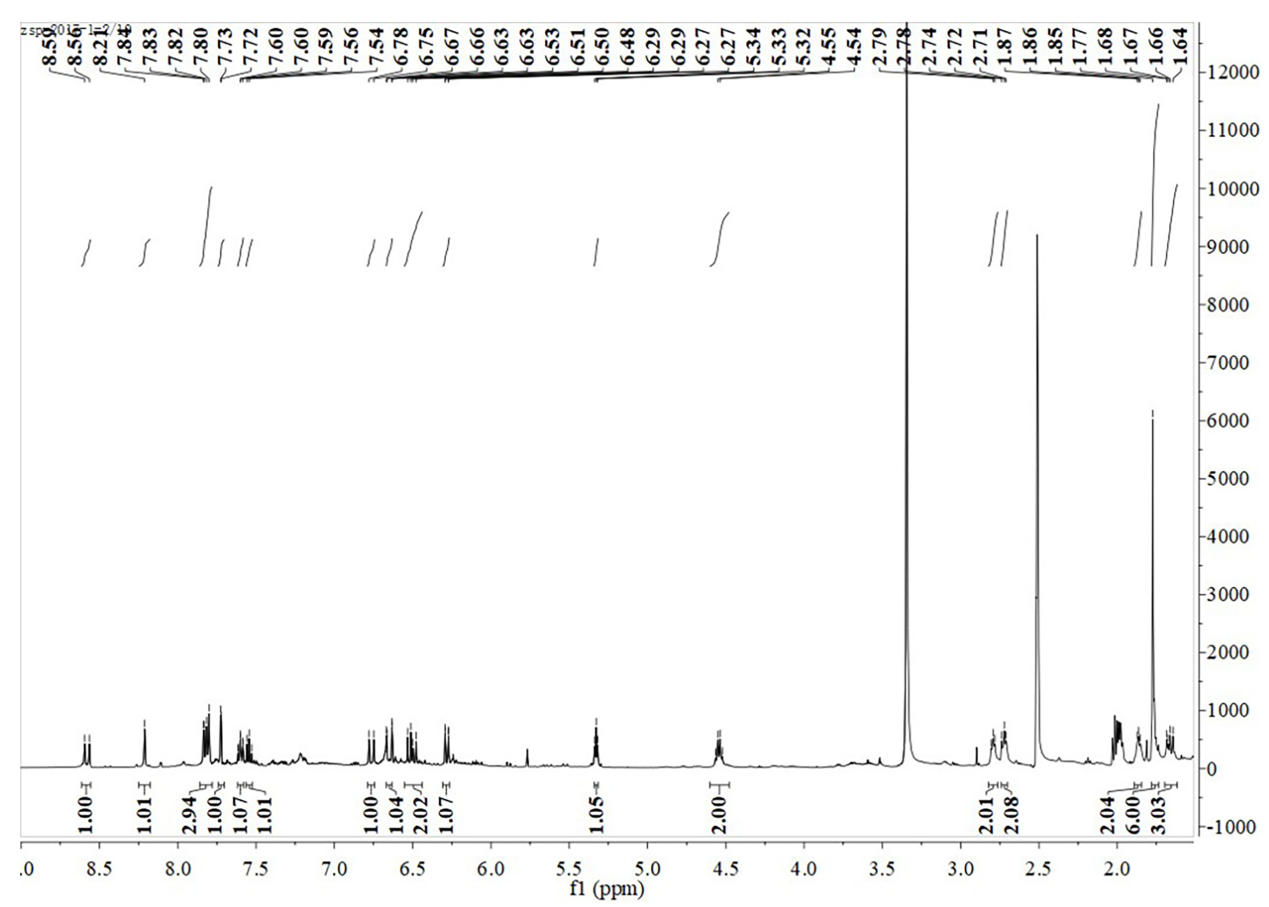


**Figure S8.** ^1^H NMR spectrum of **Cy-Cys-pH.**


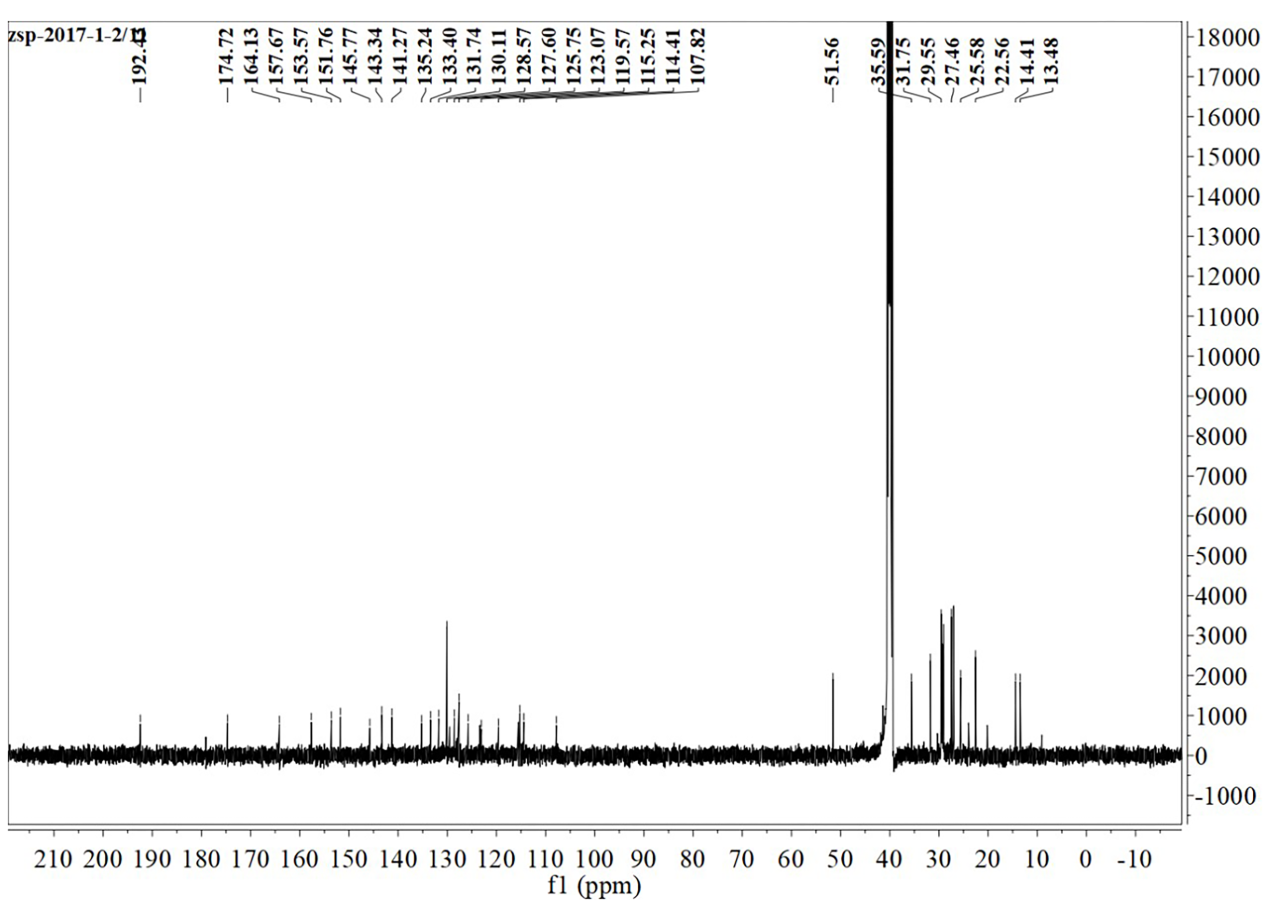


**Figure S9.** ^13^C NMR spectrum of **Cy-Cys-pH_._**


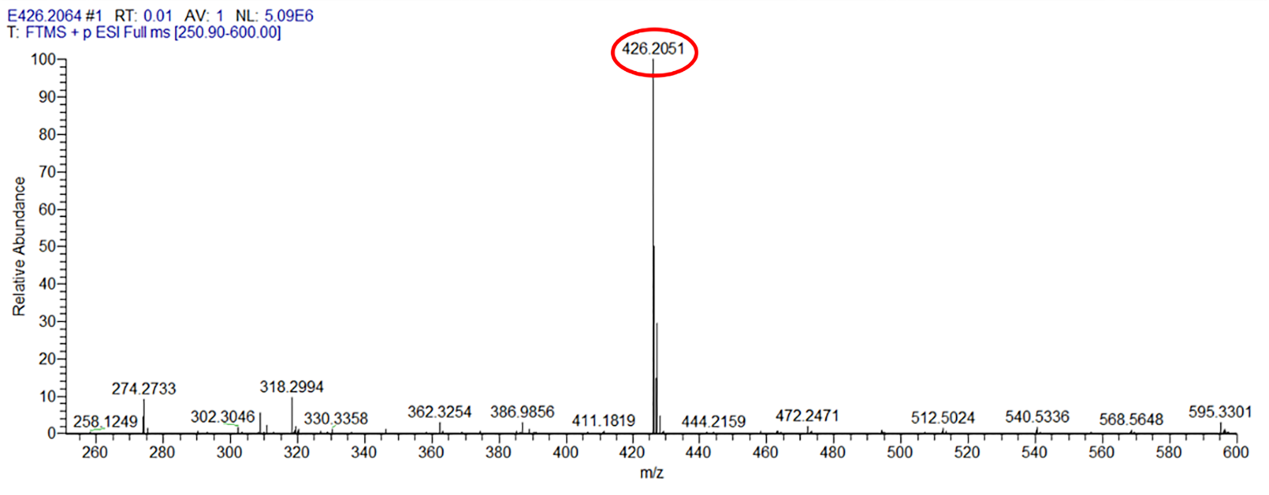


**Figure S10.** ESI-MS of the compound after **Cy-Cys-pH** reacted with Cys.


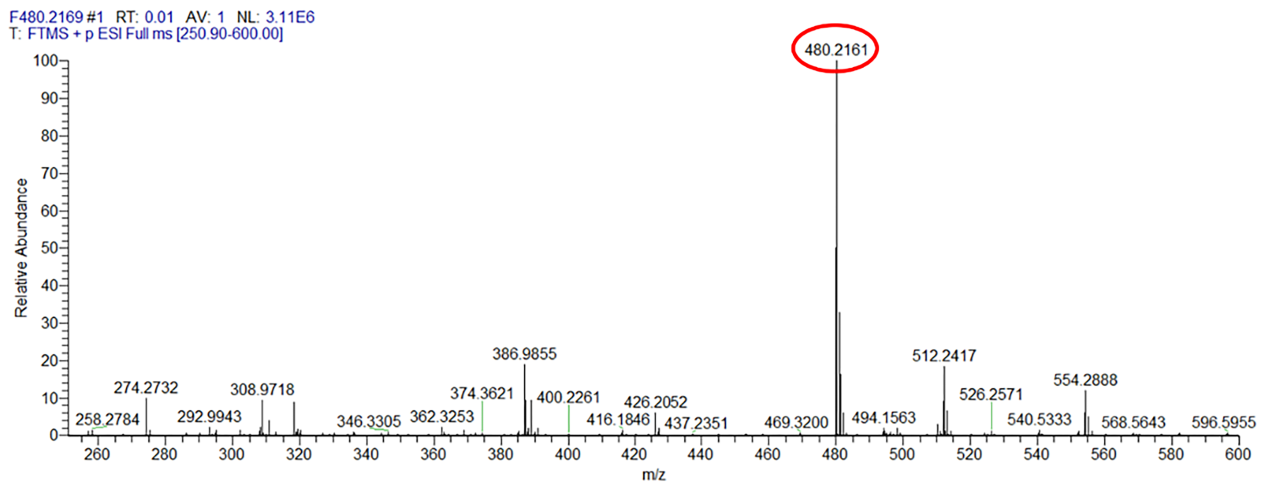


**Figure S11.** ESI-MS of compound **Cy-Cys-pH**.
